# “Slow science” for 21st century healthcare: reinventing health service research that serves fast-paced, high-complexity care organisations

**DOI:** 10.1108/JHOM-06-2020-0218

**Published:** 2021-05-04

**Authors:** Christine Jorm, Rick Iedema, Donella Piper, Nicholas Goodwin, Andrew Searles

**Affiliations:** NSW Regional Health Partners, Newcastle, Australia; School of Medicine and Public Health, The University of Newcastle , Callaghan, Australia; School of Rural Medicine, University of New England , Armidale, Australia; Centre for Team Based Practice and Learning, King's College London School of Medical Education , London, UK; NSWRHP, Newcastle, Australia; Research, Central Coast Local Health Network , Gosford, Australia; Central Coast Research Institute, The University of Newcastle , Callaghan, Australia; Health Research Economics, The University of Newcastle Hunter Medical Research Institute , New Lambton, Australia

**Keywords:** Economics, Health services, Knowledge sharing, Local area networks, Partnering, Research

## Abstract

**Purpose:**

The purpose of this paper is to argue for an improved conceptualisation of health service research, using Stengers' (2018) metaphor of “slow science” as a critical yardstick.

**Design/methodology/approach:**

The paper is structured in three parts. It first reviews the field of health services research and the approaches that dominate it. It then considers the healthcare research approaches whose principles and methodologies are more aligned with “slow science” before presenting a description of a “slow science” project in which the authors are currently engaged.

**Findings:**

Current approaches to health service research struggle to offer adequate resources for resolving frontline complexity, principally because they set more store by knowledge generalisation, disciplinary continuity and integrity and the consolidation of expertise, than by engaging with frontline complexity on its terms, negotiating issues with frontline staff and patients on their terms and framing findings and solutions in ways that key in to the *in situ* dynamics and complexities that define health service delivery.

**Originality/value:**

There is a need to engage in a paradigm shift that engages health services as co-researchers, prioritising practical change and local involvement over knowledge production. Economics is a research field where the products are of natural appeal to powerful health service managers. A “slow science” approach adopted by the embedded Economist Program with its emphasis on pre-implementation, knowledge mobilisation and parallel site capacity development sets out how research can be flexibly produced to improve health services.

## Introduction

Our world is becoming rapidly more complex with fast-moving, interconnected and outsized effects defining recent environmental, biological, social, economic and political events. This escalating complexity necessitates changes in how we study and intervene in our world ( Mitchell, 2009 ). Twentieth century research pursued large scale answers to many problems on the assumption that life and the world we live in could be, to a large extent, predictable. Recent events have shown that unpredictability is no longer the exception that proves the rule. Recent bushfires, COVID-19 and major geopolitical shifts are just three prominent examples ( Wynants *et al.* , 2020 ; Wahlquist, 2020 ; Remnick, 2016 ). These recent crises have made clear that unpredictability is not avoidable or deniable.

To deal with uncertainty in all domains, complexity must be embraced. The notion of complexity captures the idea that the future may not model itself on the past. That is, the past may offer few (and increasingly fewer) productive clues for understanding the future, even if ultimately, after much investigation and analysis, the past turns out to harbour characteristics that may be interpreted as causes underlying subsequent crises. Complexity necessitates a shift in confidence from elegant conclusions and confident generalisations about how the world works, towards an enhanced appreciation of and more local, pragmatic and democratic capability for navigating through uncertainty ( Stengers, 2018 ), a humbler attitude towards what we assume to know ( Jasanoff, 2003 ), a more tactical-strategic approach towards insufficient and absent information about the things we are needing to understand ( Simpkin and Schwartzstein, 2016 ) and a more creative perspective on increasingly fast-moving events and circumstances ( Latour, 2018 ; Klein, 1999 ).

Health services have not remained immune to this runaway complexity. Leaving events of the last 12 months aside, healthcare faces rising levels of chronic disease; more co-morbidity; runaway numbers of treatments, drugs, technologies, roles and specialties; more frequent staff and patient movements ( Britnell, 2019 ); a growing number of information sources about patients, diseases and treatments at a time when the half-life of scientific knowledge is shrinking ( Arbesman, 2012 ) due to the tens of thousands of trials that continue to update, challenge or reverse what we know; relentless expansion in the numbers of guidelines, protocols and regulations, and constantly changing (inter) organisational and financial arrangements ( Britnell, 2015 ).

Healthcare systems around the world are entering a period of transformational change. New demands related to ageing, chronicity and rising public health concerns have led to a significant move away from disease-based thinking to embrace solutions embedded in a more integrated biopsychosocial approach where care and services are coordinated around people and communities. This requires innovations that must fight health system fragmentation in favour of citizen-empowered, community-driven, coordinated and cross-sectoral solutions ( Goodwin, 2019 ). Complex problems are requiring complex service innovations.

Given these trends and developments, it is not surprising that the enthusiasm of some towards academic research, its demanding, ponderous and intrusive methodologies, and even its findings, has dwindled in recent years ( Nichols, 2017 ). In response to this sentiment as well as fuelling it, governments have for several years now kept level or even reduced funding for academic research ( Atkinson and Foote, 2019 ). Alongside this, management consultants are increasingly tasked with the development of healthcare solutions in the UK ( Kirkpatrick *et al.* , 2019 ) as well as in Australia ( Skouteris *et al.* , 2019 ), even though the increased expenditure on such consultants has been found to be associated with organisational inefficiency ( Kirkpatrick *et al.* , 2019 ).

Even before COVID-19, the problems with which people have been grappling locally are becoming more intrusive, pervasive and unsettling. The answers offered by large scale academic research nevertheless arrive late and in a format that often lacks relevance for solving here-and-now challenges and complex problems at the frontline. Research methods struggle to understand the implementation and sustainability of complex service innovations, and research funding approaches have to date not supported local innovation in ways that enables managers, clinicians and service users to tackle today's challenges ( Goodwin, 2016 ).

This paper seeks answers to the dilemmas posed by this situation. To this end, it reviews the strategies, methodologies and ideologies of prevailing research approaches that have attempted to resolve problems for staff at the frontline. The paper surmises that these approaches struggle to offer adequate resources for resolving frontline complexity, principally because they set more store by knowledge generalisation, disciplinary continuity and procedural integrity, as well as the consolidation of formal expertise, rather than by negotiating issues with frontline staff and patients on their terms and framing findings and solutions in ways that key in to the *in situ* dynamics and complexities that define frontline care.

The paper is structured in three parts. It first reviews the field of health services research and the approaches that dominate it, using Stengers' (2018) metaphor of “slow science” as a critical yardstick. The paper then shifts gear to consider healthcare research approaches whose principles and methodologies are better aligned with the principles of “slow science”. The paper next presents a description of a “slow science” project in which the authors are currently engaged, entitled “The embedded Economist” (eE).

## What is being done in the name of health services research and improvement?

Health services research and improvement endeavours fall into four camps: biomedical (“experimental science”), management; organisational and industrial theory (“safety science”, “normalisation process theory”); psychology and ergonomics (“human factors”, “theoretical domains framework”, “nudge theory”); and practice improvement studies (“action research”, “experience-based co-design”, “appreciative research”, “participative enquiry”). The first three of these endeavours concern themselves with knowledge building from experiments, measurements or objective observations. The last category is different and is addressed in the next section, as it refers to endeavours that prioritise practical change and local involvement over knowledge production.

The aim of experimental science is to test hypotheses about how the world works. Such science is critical for establishing the effectiveness of drugs or surgical interventions, among other things. Bodies and bodily reactions to interventions differ, but, by and large, biophysiology allows a considerable degree of generalisation. Experimental science has become the methodological blueprint for research in health, given its success at understanding and intervening in biophysiological and anatomical systems ( Bliss, 2011 ). This science defines most kinds of health services research and has found ways of applying its “rigorous” methods (in particular the clinical trial) to all kinds of matters, including organisational, social, behavioural and psychological phenomena. Its prioritisation of counting and numbers betrays its faith in general laws and abstract expertise at the expense of innovation necessitated by *in situ* dynamics and local specificities. This science reduces complexity for its “rigourous” procedures to continue to produce elegant but de-contextualised conclusions ( Neuman *et al.* , 2014 ).

One more recent entrant seeks to straddle all four camps for the sake of accomplishing the implementation of scientific conclusions. Implementation science pursues: explanations about why particular interventions work or do not work ( Palinkas *et al.* , 2015 ); the identification of causal factors and their interactions ( Bierbaum *et al.* , 2020 ); and the development of “tools” that serve to structure interventions, measure progress and evaluate outcomes ( Parmelli and *et al.* , 2011 ). With regard to implementation science theorisation, a popular approach is the definition of an overarching or “meta” framework, produced from the collation and at times expert consensus of implementation science's frameworks, models and theories. Frequently quoted examples are Nilsen (2015) ; Colquhoun *et al.* (2014) and Powell *et al.* (2015) , articles which offer up meta-generalisations about what works in the domain of evidence translation into practice. The core assumption underpinning this work is that, complexity notwithstanding, “all methodologies must be incorporated to fit one concise overriding implementation approach” ( Boulton *et al.* , 2020 , p. 8).

One such theorisation is normalisation process theory (NPT) which offers a “consistent framework” for the description and measurement of implementation “potential” ( Murray *et al.* , 2010 ). More recent incarnations have extended NPT in order for it to account more explicitly for complexity, but this has not dented NPT's faith and investment in meta-generalisation as the answer *par excellence* to rising healthcare complexity ( Boulton *et al.* , 2020 ). Mining a similar vein, the Theoretical Domains Framework (TDF) defines the field of behaviour change with specific focus on the identification of significant factors and techniques that correlate with measurable behavioural effect ( French *et al.* , 2012 ). Here too, the priorities are inclusivity and coherence of an increasingly wordy framework, in effect sacrificing attention to the multiplicity and complexity of the phenomena that are at issue here *practically* .

In the spirit of NPT and TDF, the dash for theoretical edification comes to a head in a recent article proposing “translational mobilisation theory” (TMT) to inform “care trajectory management” (CTM) ( Allen, 2019 ). TMT is said to relate to CTM in so far as “[m]aintaining trajectory awareness involves the translational mobilisation mechanisms of reflexive monitoring, sense-making and object formation” ( Allen, 2019 , p. 769). William James' comment from a century ago comes to mind, as for these endeavours “the key must be sought in the shape of some illuminating or power-bringing word or name. That word names the universe's principle, and to possess it is after a fashion to possess the universe itself” ( James, 1907 , p. 52). Just as NPT's self-description establishes its organisation-level preoccupation as the normalisation of particular service adaptations, and TDF's self-description heralds its commitment to the categorisation of psychological “domains” that are deemed to play a “deciding” role in the success of behavioural interventions, so TMT/CTM's self-descriptions anchor it to what is ultimately a mechanistic perspective on “how collective action is mobilised” ( Allen, 2019 , p. 768). Each of these endeavours give precedence to naming, framing and labelling their assumptions and conclusions about how the world works: intervening in situated complexity remains secondary to seeking to “possess the universe”.

A somewhat more pro-active enterprise is that of human factors (engineering), an approach that emerged from the intersection of psychology, ergonomics and engineering. Human factors (HF) research targets the ways in which humans commonly behave and think in order to adjust contexts such that they compensate for and minimise human shortcomings, rendering people's errors and mishaps less likely or impossible ( Dekker, 2014 ). Like NPT and TDF, this approach is equally theory-heavy. Anchoring its expertise to (abstract) claims about cognitive deficiency and human resilience, HF's priority is to scale up its “what-works” solution designs without considering that such designs may perturb or even undermine the local ecologies of sites' existing practices. Again, the dynamic complexity of local care practices, that is, the relational, cultural, financial and political dimensions of situated care are pushed into the background. With this, the *in situ* achievement of reform and the *ongoing* negotiation of improvement become ancillary concerns.

Health services research (HSR) is thus replete with endeavours that prioritise academic-disciplinary abstraction over local-practical complexity, notwithstanding all the theoretical heat about complexity as lens for improvement and as guide for reform (e.g. Leykum *et al.* , 2014 ; Paley, 2010 ; Greenhalgh *et al.* , 2010 ). HSR's preoccupation with disciplinary theorisation over local-practical achievement is not entirely surprising: it has always sought to distinguish itself from quality improvement (QI) as “merely local”. The logic that operates here is that “real” research requires objectivity (distance) and knowledge generalisation (abstraction), whereas QI just concerns the “translation” of that knowledge into what people do at work, as they try to solve local problems. This logic is disabling for both HSR and for QI. It is disabling for HSR by dissociating it from the practical dilemmas and choices faced by practitioners and patients. It is disabling for QI by relieving QI from the onus that bears on any endeavour seeking to reform health care; that is, the imperative to theorise about its processes and outcomes “so as to provide for [later] discoveries” (Peirce, cited in Dewey, 1938 , p. 9).

## Serving local practices and services

The endeavours, thus, far reviewed prioritise knowledge in the form of “elegant truths” about how and why people (should) do the things they do, and about how and why interventions need to be structured to be effective. This knowledge is “elegant” in so far as that its pronouncements are intended to be universally valid and adopted by being “translated” into the languages and structures of local practice. This knowledge is assumed to have attained the standard of objectivity, where objectivity stands for researcher-researched distance, methodological and analytical replicability, and results in generalisability. Its translation into practice faces the hurdle of situating knowledge produced in one place in another place that is quite unlike its place of origin. What defines the place of application is that it is real world, local and most likely messy. By having deferred engaging with the messiness of reality until it gets to the point of knowledge *translation* , elegant knowledge has in effect exempted itself from negotiating its contents with end users, from adapting itself to complex situations, from reinventing itself in response to emergent problems and from having to acknowledge that local practices embody their own ecologies and their own wisdoms.

An investigation of how improvements and innovations are translated and “diffused” within and between service organisations concluded that there was a lack of any robust understanding in how innovations can be implemented and sustained across contexts and settings ( Greenhalgh *et al.* , 2004 ). This work poses a challenge for future research and practice that remains to this day. Historical attempts have used a blend of realistic synthesis, behavioural theory and mixed-methods to foster understanding (e.g. May *et al.* , 2016 ). However, these too have led to rather passive and descriptive interpretations that explain how things were.

In order to break through into the local and practical realities inhabited by frontline actors (clinicians, managers and patients), research thus needs to attenuate its experimental, objectifying and standardising priorities and preoccupations and invent ways of connecting with local problems and actors that go beyond aspirational claims about engaging with “work as done” ( Hollnagel, 2015 ). A range of approaches have taken up this challenge: action research ( Reason and Bradbury, 2008 ), appreciative enquiry ( Cooperrider and Whitney, 1999 ) and participative research ( Park, 1999 ), among others. The principles that unite these endeavours are threefold: communally conducted and locally targeted research engages strategically and pragmatically with the complex dynamics of *in situ* practice and relationships; participation by local stakeholders in this research is critical for refining the questions asked, for adapting the research process to local challenges and for targeting outcomes at what matters to those who are to benefit from the knowledge produced; and, last and most significantly as well as least apprehended, these researcher-researched dynamics and activities harbour the seeds of crucial kinds of more general and theoretical knowledge ( Iedema *et al.* , 2013 ).

One, particularly, interesting example of such research is experience-based co-design or EBCD ( Bate and Robert, 2007 ). EBCD is a type of co-production that involves practitioners, service users and anyone else with a stake in care in co-designing solutions for local problems. Co-production is defined somewhat more broadly as “a process through which inputs from individuals who are not [generally] ‘in’ the same organisation are transformed into goods and services” ( Ostrom, 1996 , p. 073 cited in Beckett *et al.* , 2018 ). The idea behind co-production is that those for whom research knowledge is meant to matter and be useful, in this case, health service staff and patients are active agents, rather than passive recipients, and that their knowledge is as valued as researchers' knowledge ( Heaton *et al.* , 2016 , cited in Beckett *et al.* , 2018 ). Staff and patients become co-investigators and are granted a say in the selection of research questions, data decisions, analytical processes and interpretations ( Iedema *et al.* , 2013 ). Both researchers and stakeholders bring their unique expertise (methodological, contextual, experiential, relational-political and topic related) to the project to generate research findings.

While a popular approach ( Palmer *et al.* , 2019 ), co-design is limited in its application due to the propensity of research funding agencies to require up front complete study designs setting out hypotheses, process plans, outcomes and benefits. Indeed, co-design's indeterminacy has been described in a recent publication as “the dark side of co-production” that does “damage to interpersonal or organisational relationships, research careers and researcher independence and credibility” ( Oliver *et al.* , 2019 ). These authors' complaints are instructive about “business-as-usual” HSR and its presumptions:

Under business-as-usual rules, researchers spend their time identifying genuine and novel gaps in the knowledge base, which have to be justified at length to colleagues and funders. However, the coproduction process can lead to researchers being asked to answer questions which are dull, not novel (little contribution to the scientific literature), or not generalisable (focused on local issues) – and therefore not easily publishable. ( Oliver *et al.* , 2019 )

In seeking to protect “business-as-usual” HSR, Oliver and colleagues sacrifice the interests, needs and preferences of practitioner and patient stakeholders, while nevertheless gesturing at the problem of “metrics and funder priorities [that] can often be disconnected from public value and egalitarian imperatives” ( Williams *et al.* , 2020 ).

In contrast to co-design, video-reflexive ethnography or VRE is an approach that fully foregrounds the dynamic complexity of *in situ* practice ( Iedema *et al.* , 2019 ). VRE nurtures local learning and generates learning dynamics across a site. Researcher-practitioner-patient collaboration is fundamental to its investigative processes, which centre on people reviewing real-time video footage of their *in situ* practices and experiences as a means to engendering deliberation and reflection about existing systems, practices, understandings and relations. While generally funded to address everyday care challenges such as arising from infection risk ( Gilbert *et al.* , 2020 ; Hooker *et al.* , 2020 ), dying ( Collier *et al.* , 2016 ) or cross-professional handover ( Iedema *et al.* , 2012 ), VRE takes situated complexity (emotional, interpersonal, political, pragmatic, technical, intersectoral, etc.) as its investigative point of departure and as its ultimate source of continued learning for both researchers and participants.

Taking the principle of collaborative enquiry another step further, Marshall and colleagues advocate for “embedded researchers” or researchers-in-residence ( Marshall *et al.* , 2014 ). The researcher's embeddedness involves her in the complexities of everyday practice and decision-making. A method germane to this work is ethnography, allowing for the detailed tracking of complexities. Vindrola-Padros and colleagues (2017) identified the following success factors for embedded research:

1. *Researcher immersion in the site* to understand the context, site aims and the pressures faced at different levels, in order to tailor strategies (2017, pp. 71–72).
2. *Development of relationships with local teams* – to uncover different viewpoints *,* promote ownership and anticipate potential tension produced by competing views (2017, pp. 72–76). Regular meetings with teams and management are important to provide iterative feedback and maintain relationships. (2017, p. 77)
3. *Critical reflection to* help the researcher maintain a clearer idea of their role and capacity to intervene (2017, pp. 76–77).
4. *Capacity building* which includes teaching skills to site participants, for instance, in evaluation: “In contrast to other research approaches that tend to be based on the development of individual partnerships between researchers and staff, the embedded approach centres on the incorporation of research into the organisation's systems, processes and practices, thus promoting its sustainability over time.” (2017, p. 77).

Cheetham and colleagues ( Cheetham *et al.* , 2018 , p. i68) call attention to the need, when undertaking embedded research to “scale back expectations about potential impact and recognise the significance of incremental attitudinal change, leading to a willingness to try different ways of working”. Recognising the critical role of relationships, trust and emotion, Pain and colleagues note that “the emotional dimensions of co-production are not side-effects, but are active in generating impact: ‘ *Feelings produce impacts produce feelings”* ( Pain *et al.* , 2016 ). Collaborative research takes time as relationships are central to the work and need to be built and maintained. Yet in spite of their obvious benefits, embedded researcher arrangements do not appear to have been sustained long-term. Care organisations with thousands of staff should be able to support a few embedded researchers if they perceived them to be of value. This raises questions about services' understanding of the purpose of collaborative study arrangements more generally, of its approaches and its outcomes.

These questions may be clarified as follows. The approaches just discussed are commonly seen as germane to QI, as benefitting only isolated settings and as impacting on idiosyncratic processes unique to those settings. They thereby are often seen to disqualify themselves as *research* by failing to produce generalisations that benefit “everyone everywhere” and that emerge from scientific enquiry that assumes a “point of view from nowhere” ( Nagel, 1989 ). If the embedded researcher prioritises the latter (generalisations), he/she may be seen to fail the service in its need to solve acute problems. If the researcher prioritises the former (acute problems and local solutions), he/she may be not be seen to deserve the appellation of researcher and will appear to be replaceable by people with “improvement expertise” (such as disseminated through IHI) and who are already on staff. However, both these scenarios fail to engage with the difficulty at issue here: the continued prioritisation of what Stengers calls “fast science” over an approach to science that takes on pertinent challenges without sacrificing its commitment to knowledge building and knowledge sharing.

## Slow science

In her recent book, Isabelle Stengers characterises “point-of-view-from-nowhere” science as “fast science” ( Stengers, 2018 ). Fast science is fast as it operates as a collection of self-contained and mechanised routines, including research protocols, methodological procedures and analytical algorithms. Quite circularly, each of these are determined, controlled and conducted by the academic, funder and industrial institutions that sanction, monitor and legitimate such science. This situation, Stengers claims, has led to the disembedding or *industrialisation* of such science, automatising its operations, financing and applications. Such science is disengaged from the complex realities and everyday living circumstances about which it makes pronouncements: “The symbiosis of fast science and [the health] industry has privileged disembedded knowledge and disembedding strategies abstracted from the messy complications of this world. But in ignoring messiness, and dreaming of its eradication, we discover that we have messed up our world” ( Stengers, 2018 , loc 1940). Instead of fast-paced science, we need to slow science down.

Stengers defines slow science as inviting those to the table who have a stake in the phenomena studied, in the structuring of study processes, in the analytical approach and in the conclusions and outcomes produced from such science. Slow science sets up a symmetry, a democracy, through which researchers are able to learn from stakeholders and *vice versa* . Here, neither party resorts to “capturing” or caricaturing the other: *viz.* the researcher as expert with access to The Truth, or the stakeholder as the uninformed, confused actor in need of being enlightened by expertise. Slowing science down means that its operations and its consequences are constantly checked against and aligned with agreements about what kinds of studies are worth doing, what kinds of knowledges are worth cultivating and, ultimately, what lives are worth living:

Slowing down [science] means becoming capable of learning again, becoming acquainted with things again, reweaving the bonds of interdependency. It means thinking and imagining, and in the process creating relationships with others that are not those of capture [i.e. caricature]. It means, therefore, creating among us and with others the kind of relation that works for … people who need each other in order to learn – with others, from others, thanks to others – what a life worth living demands, and the knowledges that are worth being cultivated ( Stengers, 2018 , p loc 1,366).

Slow science therefore implies that research and evaluation needs to take on a more practical and participatory form to support continuous learning. This is particularly important where there is a need to improve or change care systems, but where a lack of evidence supports any one particular path or trajectory. Reflexive practice requires not only a more intimate relationship between research and practice in which research needs to play the supportive (but not subordinate) role to practice but also where practice should respond to research findings in equal measure ( Goodwin, 2019 ).

## “Slow science” in practice: “the embedded economist”

An example of slow science is “The embedded Economist” Program. Economic evaluation is a way of doing research that is increasingly utilised by health services to ensure research recognises that decisions have resource implications. Once resources are used for a given activity, they are forgone. Decisions in healthcare can have long-term impacts on health outcomes and economic activity; they can create or remove waste in healthcare. It's a science that curates and creates evidence for decision-making. It is centred on providing information on the efficiency of interventions ( Brousselle and Lessard, 2011 ). Technical efficiency results when benefits are maximised and opportunity costs minimised. Common types of analysis are: cost-minimisation analysis, cost effectiveness analysis, cost utility analysis and cost benefit analysis. Many economists are also impact evaluation experts.

The use of economic evaluations at the *local decision-making level* in healthcare remains limited ( Searles *et al.* , 2018 ; Baghbanian and Torkfar, 2012 ; Eddama and Coast, 2008 ). This is problematic because most of Australia's health budget, 70%, is spent at the “local level” through hospitals, community care and through primary care ( Searles *et al.* , 2018 ; Australian Institute of Health and Welfare, 2017 and Lessard *et al.* , 2010 ). The number of published economic evaluations applied to the field of improvement and implementation research is, however, modest ( Roberts *et al.* , 2019 ). One of the reasons for this is that the design of economic evaluations looking at implementation must embrace scenarios that are complex, multi-dimensional and evolving. Yet existing methodological design tends to “screen out” complexity, meaning that economic evaluations are likely to fail in providing answers to today's health system challenges. Some more recent innovations – such as cost-consequence analysis and multi-criteria decision analysis – are seeking ways to address this, but economic evaluations of the future must adapt better to support decision-making ( Tsiachristas *et al.* , 2016 ).

The *attitude* of decision-makers towards the usefulness and necessity of economic evaluation in informing decision-making processes is largely positive ( Hoffmann *et al.* , 2002 ; Lessard *et al.* , 2010 ; Roseboom *et al.* , 2017 ). Despite this, the literature highlights a plethora of barriers ( Hoffmann *et al.* , 2002 ; Roseboom *et al.* , 2017 ; Zechmeister-Koss *et al.* , 2019 ; Brousselle and Lessard, 2011 ; Williams *et al.* , 2008 ); among others, the applicability and responsiveness of economists and economic evaluations to decision-makers' needs and context, and communication of the results in a way that is meaningful for decision-makers.

Although it sounds like a very applied field of research, Ross (1995) gathered complaints about communication in the form of the “jargon” of economists and about “academic” health economists seeming to place more emphasis on the rigour of their methods than on communicating the principles involved to the decision-makers. Troublingly, Brouselle and Lessard (2011) speak of some barriers being the results of a “paradox” in the evolution of economic evaluation: Whilst there has been an increase in insight into the need for economic evaluations, its influence in practice has become more limited ( Drummond, 2004 ; Eddama and Coast, 2008 ; Williams *et al.* , 2008 ). More specifically, evolving methodological sophistication has meant there is a decrease in capacity to use it.

One solution put forward to address these barriers is the need for health economists to engage with health services to better understand their concerns, rather than focusing on a more academic and/or transactional approaches to economic evaluation. Several scholars ( Buxton, 2006 ; Hoffmann *et al.* , 2002 ; Hoffmann and von der Schulenburg, 2000 ; Zwart-van Rijkom *et al.* , 2000 ; Zechmeister-Koss *et al.* , 2019 ) argue for the need for an active communication process before research starts.

For evidence from economic evaluation to be used in healthcare decision-making, it needs to be *acceptable and accessible* ( Merlo *et al.* , 2015 ). Acceptability depends on the accuracy and validity of research methods, the relevance given institutional structures and ethical concerns. Accessibility depends on the timeliness of the research, the quality of communication and decision-makers' level of understanding of economic evidence ( Merlo *et al.* , 2015 ). The fact that decision-makers often lack the knowledge to engage in a meaningful way has led to calls to educate and build their capacity in economic evaluation ( Eddama and Coast, 2008 ; Roseboom *et al.* , 2017 ; Zechmeister-Koss *et al.* , 2019 ; Williams *et al.* , 2008 ). Responding to these challenges, the embedded Economist Program embodies a radical extension of the principles of co-design. Radical, because research topics will be chosen by the health service, so decision-making power sits with them, and techniques are negotiated to enhance shared understanding and transfer of economic knowledge (including by an associated education offering).

We selected to “embed” economic evaluation because health services, despite the enormity of the economic decisions they make, the financial pressures they work under, the large percentage of the GDP they consume and the high amount of waste that has been identified, rarely have access to internal health economic advice ( Searles *et al.* , 2018 ). In addition, until very recently, economic evaluation has not been a prominent part of health research nor of improvement research, especially at the local level ( Roberts *et al.* , 2019 ).

Learning from the overall failure of health services to embrace research, the co-design literature and the call for “slow science”, the distinctive features of the embedded Economist Program are as follows (see Table 1 ). A prolonged pre-implementation phase is incorporated to establish relationships and trust. The researcher is embedded in the management chain as well as with program, service and clinical teams. The researcher (the economist) is not undertaking analyses – research acts – with the intention of publication (some might be published, but only if this is a health service objective). Thus, novelty (essential for successful peer reviewed publication) is not a criterion for undertaking work and the researcher is freed to do the research the health system desires/requires; “your problem is our research” or “your problems are why we do research”.

Further, the researcher is an economist (rather than an ethnographer or sociologist). Their research uses numbers and values resources in financial terms. This is the currency of management – who are always auditing, meeting targets, etc. Their research needs are/often monetary in focus – cost is a pressure point for all health decisions and systems (even more so post-COVID-19). The economist/researcher also encapsulates a number of “role domains” necessary for effective knowledge mobilisation including: information manager, linking agent, capacity builder, facilitator and evaluator ( Glegg and Hoens, 2016 ; see too Churruca *et al.* , 2019 ; Vindrola-Padros *et al.* , 2017 ; Wye *et al.* , 2020 ; Kislov *et al.* , 2014 ). The evaluator domain refers to assessing context, processes and outcomes ( Glegg and Hoens, 2016 , p. 120) – an area of expertise for all the economists we embed. The ability to evaluate means “barriers and facilitators of evidence use at the individual, team, and organizational level” are identified and “the most promising strategies to support the creation of shared knowledge and its subsequent application” can be selected ( Glegg and Hoens, 2016 , p. 120). The embedded work they undertake with organisational teams then *harnesses the socialisation of knowledge,* whereby knowledge generates social networks by being developed from social networks ( Brown and Duguid, 2000 cited in Wye *et al.* , 2019 ).

## Concluding discussion

This article has identified some persistent problems in health services research and care improvement. One prominent problem identified was the inflation of research as an activity that becomes a source of ultimate truth; an ideology that equally props up the *status quo* of formal research expertise. As “fast science”, such research fails to spend sufficient time on learning not just about but also learning from practice and from stakeholders. It expects and instructs stakeholders to behave in specified ways and then elevates the problem of why its advice does not take root in practice as yet requiring more (implementation) “science”, perpetuating the problem on a different front. The priorities of distance, predetermined analytical methods, generalisability and replicability are precisely the reasons for the present impasse in healthcare improvement. In domains where complexity outruns our ability to formulate rules and knowledge, conventional research continues to operate largely to safeguard its own disciplinary priorities, rather than acknowledge its limitations and the need to engage in a radical paradigm shift towards “slow science”.

The shift towards “slow science” needs to take place at different levels. Managers, practitioners and patients need to be positioned as intelligent players and as critical to any knowledge that is developed about how to go forward. The centres of knowledge production will move to where care happens – the service, the ward, the specialty. The priority of research should not in the first instance be “to know”, but “to engender opportunities for learning collectively and to engender collectives” confidence to learn'. Knowledge now needs to be flexibly produced among those who want to have a stake in that knowledge, and in where that knowledge then takes us. Knowledge needs to be increasingly produced here and now (in so far as the organisation and planning of care is concerned) because complex circumstances resist “known knowledge” and demand emerging (newly renegotiated) knowledge that is refracted by the complexities of the present. Such knowledge becomes possible if we all learn to communicate about complex circumstances in ways that no longer privilege specific lenses, expertises, analyses, but in ways that provide opportunities for blending and entangling approaches, interests, concerns and ideas ( Stengers, 2018 ).

The embedded Economist Program exemplifies these principles of slow science. The progam's priority is the negotiation of such knowledge as what matters to local actors and the articulation among different expertises and disciplines. Such processes take time and are hard pushed to conform to predetermined hypotheses, plans, or outcomes. In these and other ways, the embedded Economist Program, in particular, and slow science in general are out of sync with the values and norms that currently define HSR (funding). The complexities of the contemporary world are such as to necessitate slowing the science of healthcare reform and improvement right down, embedding it in practices and with stakeholders, complementing expert-based evidence and disciplinary hierarchies with local learning and self-organising improvement dynamics “from within” ( Iedema *et al.* , 2013 ). A slow science paradigm shift is paramount for reviving the relevance and pertinence of health services research lest its assumptions, processes and outcomes continue to be met with rising resistance and scepticism.

## Figures and Tables

**Table 1:** The embedded Economist Program

An intervention being implemented by [de-indentified]
*Overview*
In line with the concepts set out above the intervention involves Provision of learning support in the form of an online university course and a community of practice, both focused on economic evaluation and available free of charge to participants from the research sites and Provision of a health economist to work with six health service research sites for three months at a time (each preceded by a long pre-implementation phase) Together, these interventions are known as the “embedded Economist” Program (eE)
*Aims of the eE program*
To increase health service staff awareness of the benefits of economic evaluation To develop health service staff knowledge and capacity to access and apply economic evaluation principles, methods and tools in decision-making through formal training and extended exposure to an embedded economist To facilitate health service practice change and the routine application of economic evaluation principles in decision making
*Aims of the eE evaluation*
To evaluate the contextual, procedural and relational aspects of embedding an economist within health service To capture the outcomes and impact of embedding an economist within health services and providing specialist economic evaluation education
